# Three-Dimensionally-Printed Joint-Preserving Prosthetic Reconstruction of Massive Bone Defects After Malignant Tumor Resection of the Proximal Tibia

**DOI:** 10.7759/cureus.13784

**Published:** 2021-03-09

**Authors:** Onur Gursan, Mustafa Celtik, Berkay Yanik, R. Bugra Husemoglu, Hasan Havitcioglu

**Affiliations:** 1 Department of Orthopedics and Traumatology, Dokuz Eylül University, Izmir, TUR; 2 Department of Biomechanics, Dokuz Eylül University, Izmir, TUR

**Keywords:** 3d-printed instruments, malignant tumor resection, customized instruments, patient specific prosthesis, massive bone defects, joint preserving reconstruction

## Abstract

Joint-preserving prosthetic reconstruction for massive bone defects has the potential to be a new and revolutionary treatment option. In this paper, we discuss the case of a 30-year-old female patient who presented with pain and swelling around the knee for three months. The patient underwent this procedure. Postoperative patient satisfaction, pain scores, and range of motion results were found to be promising. We believe that this method has the potential to be the next stage in the quest for better treatment options for this condition.

## Introduction

Three-dimensionally (3D)-printed joint-preserving prosthetic reconstruction after malignant tumor resection has the potential to be a new treatment option for a select group of young patients. Conserving the articular surface and ligaments can preserve the normal kinematics of the knee, rather than trying to mimic it. Precisely reconstructing the anatomy of the patient can result in a better sense of proprioception and range of motion [1,2]. Careful dissection and uttermost respect for the soft tissue and the vascular anatomy of the knee are the keys to the viability of the articular surface and the surrounding structures. 

Reconstruction of massive bone defects after tumor resection poses a challenge for orthopedic oncologists. Among the reconstruction options for massive bone defects after tumor resection, allografts [3], autografts, or mega prosthetic arthroplasty [4] are the first options that come to mind. However, these options are not cost-effective, and can also cause patient dissatisfaction due to infections, loosening, and implant incompatibility. Patient-specific implants minimize the risk of incompatibility, loosening, patient dissatisfaction, and the need for revision surgery, which may reduce the financial burden on the healthcare system.

In this report, we share our preliminary experience with the 3D-printed joint-preserving prosthetic reconstruction and aim to emphasize the importance of protecting the ligaments and the articular bone component of the joint. This method of treatment has the potential to be a major weapon in the surgeon's arsenal as a salvage procedure in young patients.

## Case presentation

A 30-year-old female with no conclusive history of trauma presented with progressive complaints of pain and swelling in the proximal of the posterior region of the knee for three months. Our physical examination and subsequent imaging of the affected extremity revealed a cortex-destructing lesion located at the posterior part of the proximal tibia. Clinical examination revealed a tender palpable lump located below the popliteal area approximately 4 x 2 x 2 cm in size, with no overlying erythema. No signs of local infection were present.

The patient did not have any predisposing risk factors such as smoking or alcohol consumption. Her general well-being and vitals were stable. She was diagnosed with familial Mediterranean fever (FMF) and was prescribed colchicine. She had no surgical history and no known medication allergies.

Her laboratory test results were within normal limits: WBC: 7,800/mm^3^ (64% neutrophils, 29% lymphocytes, and 5% monocytes), hemoglobin: 13.6 g/dL. C-reactive protein was 3.3 mg/L (normal level: <5 mg/L), and erythrocyte sedimentation rate (ESR) was 11 mm/h (normal range: 0-20 mm/h). Alkaline phosphatase (ALP) was 93 U/L (normal range: 30-120 U/L), and lactate dehydrogenase (LDH) was 170 U/L (normal range: 125-220 U/L).

**Figure 1:**
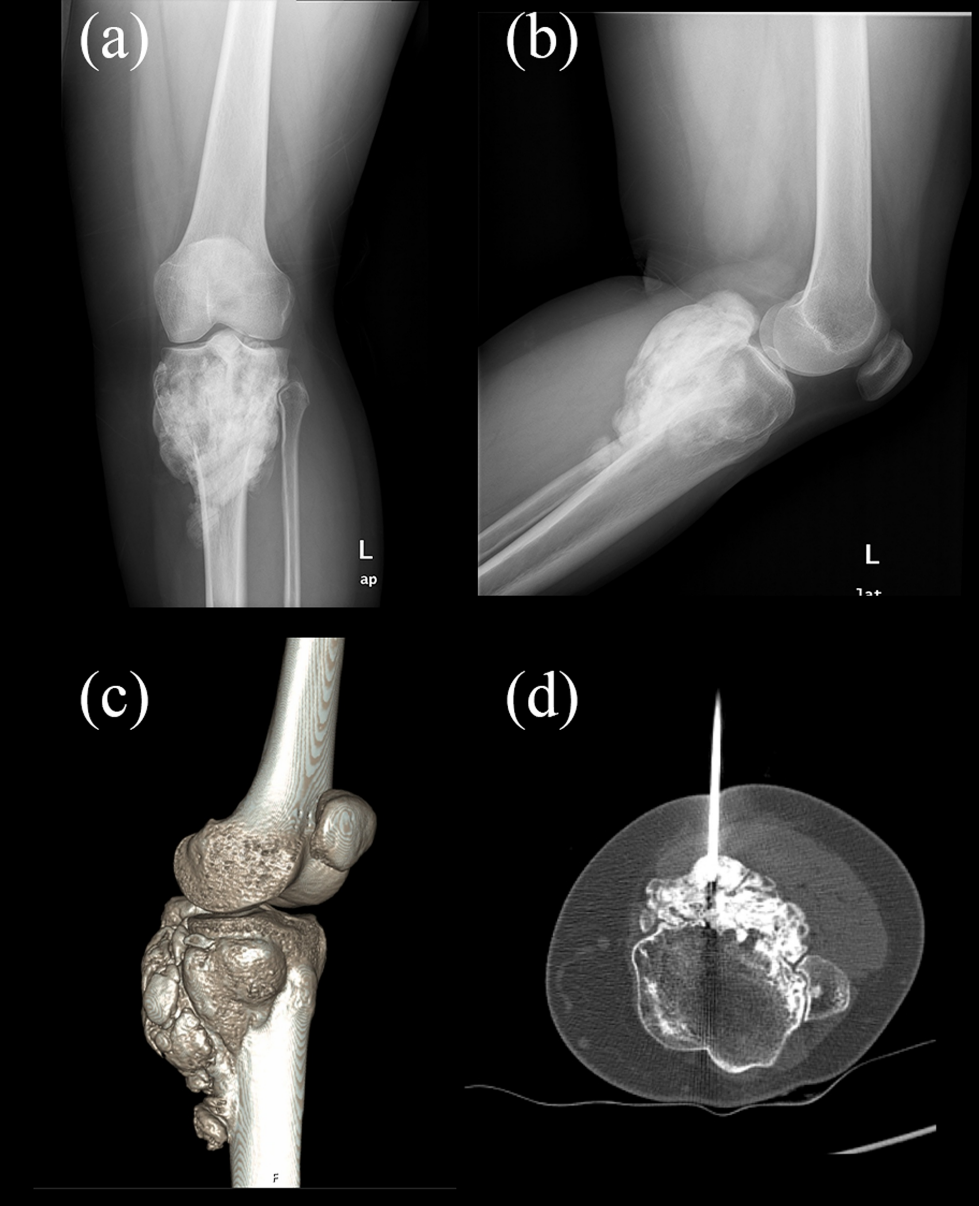
Preoperative imaging of the patient (a-b) Preoperative X-rays; (c) 3D CT image; (d) CT-guided percutaneous biopsy CT: computed tomography

The X-ray imaging showed a solid cortex-expanding mass located at the proximal tibia (Figures 1a, 1b). CT scan revealed a diffuse calcific component surrounding the bone structure, reaching dimensions of approximately 8 x 2.5 x 11 cm (Figure 1c). No lytic or destructive changes were detected in the fibula. Positron emission tomography (PET) scan showed blood flow increase and hyperemia in the 1/3 proximal part of the left tibia. In late static and single-photon-emission CT (SPECT)/CT images, increased osteoblastic activity was observed. These findings suggested malignant processes. A CT-assisted biopsy was performed under sedation; a local anesthetic agent was administered to the mass of the left tibia. Guided by the CT images, biopsy material was obtained using a biopsy needle (Figure 1d). No complications were observed after the procedure.

Materials obtained were evaluated by a specialized musculoskeletal pathologist; 9-mm tru-cut biopsy materials were processed. Histopathological features and MDM2 positivity in some of the spindle-fibroblastic cells suggested the potential for conditions such as "low-grade osteosarcoma" and "parosteal osteosarcoma".

Preoperative planning

**Figure 2:**
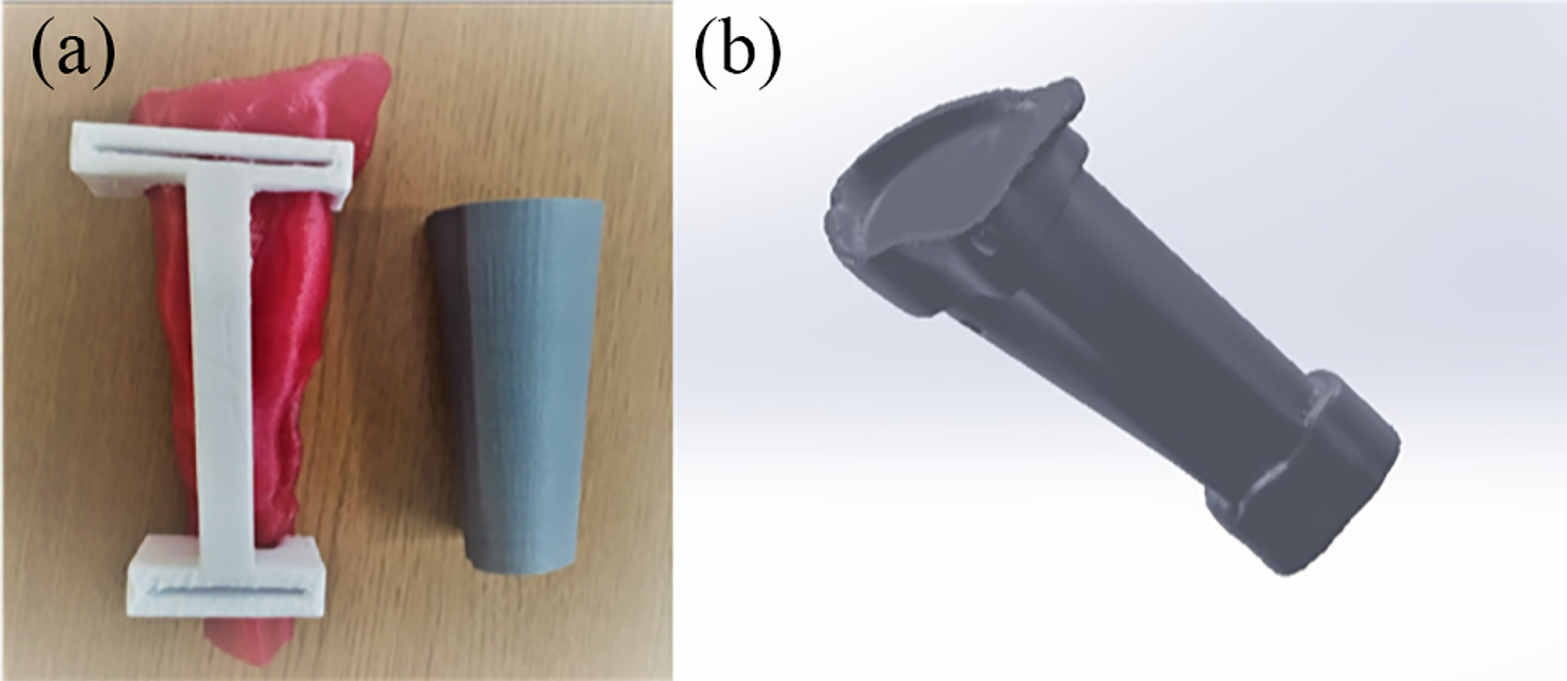
Preoperative planning (a) Preoperative planning and the design process of the patient-specific cut guides; (b) virtual 3D design process of the patient-specific joint-preserving prosthesis

Dual-stage reconstruction was planned. The aim of the preliminary surgery was to successfully preserve the epiphysis of the proximal tibia while attaining a tumor-free tibia. To perform the ideal resection of the tumor, 3D modeling and cut guides were created before the operation (Figure 2). The aim of the second surgery was to evaluate the viability of the preserved epiphysis and reconstruction of the bone defect using the 3D-printed design, which was augmented with hydroxyapatite coating to enhance the osteointegration of the implant.

Preliminary surgery

Firstly, with the aid of radiological examinations and histopathological diagnosis of the biopsy, the lesion of the patient was staged and the indications for resection were confirmed; 3D CT scans of the bilateral lower extremities were performed to obtain a surgical margin negative wide resection. MRI scan was performed to detect tumor borders and surrounding reactive tissue. For these examinations, the parameters applied by the Dokuz Eylül University Hospital Department of Radiology (1-mm cross-section and matrix of 512 x 512) were used and scans were made accordingly with these parameters. Then, the image was saved in the Digital Imaging and Communications in Medicine (DICOM) format and transferred to the 3D Slicer software for the reconstruction of 3D geometric models. Virtual mechanical analysis of the solid model created was performed. According to the shape and structure of the tumor, and by using the Meshmixer 3.5 software (Autodesk, Inc., Mill Valley, CA), osteotomy guides were 3D-printed using polylactic acid (PLA). These guides ensured that the tumor was completely removed.

**Figure 3:**
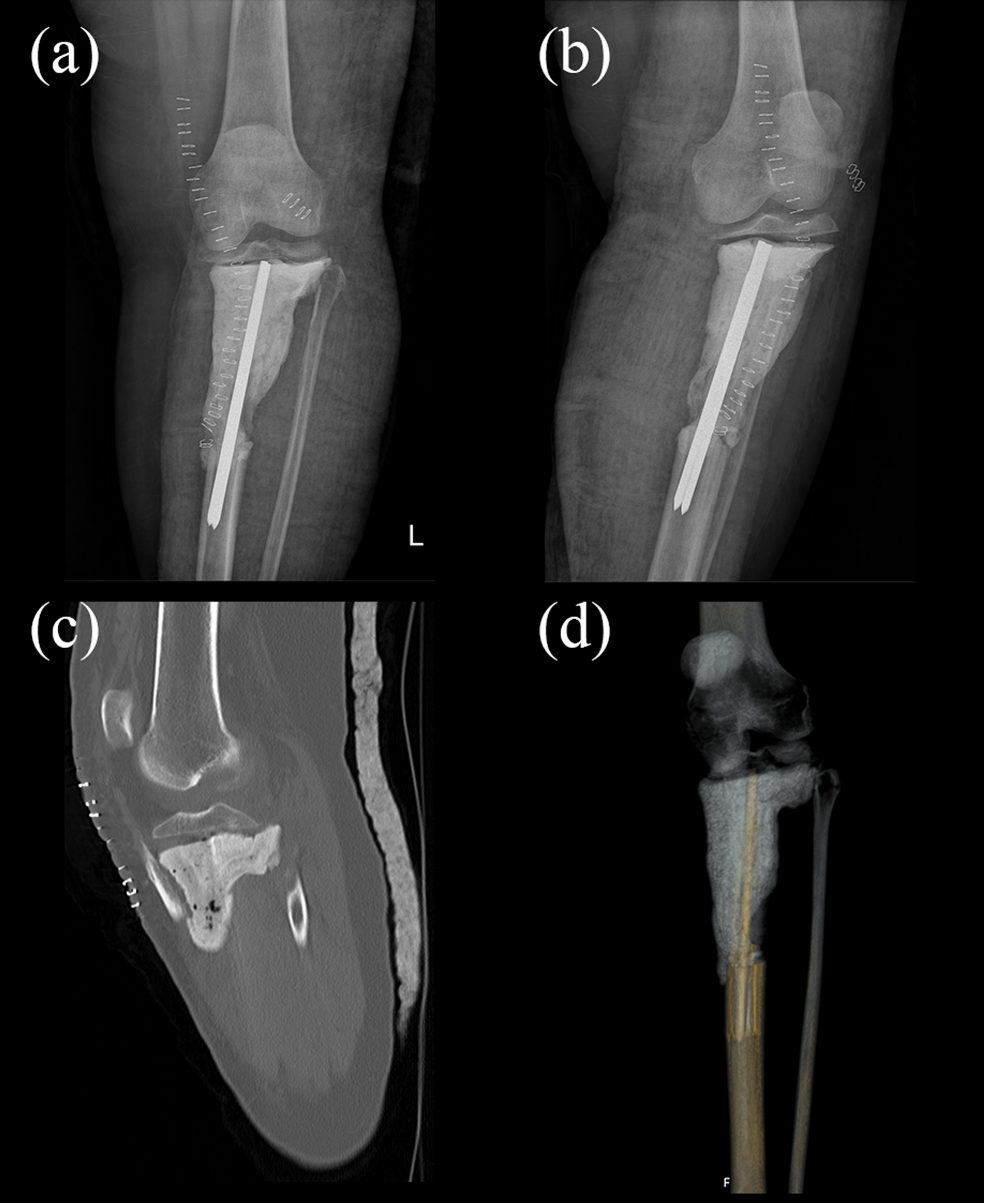
Postoperative imaging of the patient (a-b) Postoperative X-rays; (c,d) postoperative CT images CT: computed tomography

After the necessary preparations, the operation was performed. An approximately 20-cm long longitudinal incision was made at the midline of the knee, extending from the superior pole of the patella to 10 cms below the tuberositas tibia. The biopsy tract was excised during the first stage of this procedure. Arthrotomy was performed to check the viability of the anterior and posterior cruciate ligament and the menisci. Tibial tubercle osteotomy was performed. A tumor-free 5-cm segment of the bone that included the tibial tubercle and a portion of the anterior crest of the tibia was elevated. Prothesis design houses an indentation for this bone fragment to be fixated. The tumor was removed successfully using 3D-printed resection guides. To preserve the length of the limb and to avoid complications like infection, a hand-made cement spacer was used. Preserved epiphysis of the proximal tibia was then relatively stabilized onto this spacer using a conventional tibia intramedullary nail (Figure 3). After the operation, the patient had no medical problem at her follow-up and was discharged with the appropriate postoperative care recommendations. Pathology later confirmed their preoperative diagnosis as ‘parosteal osteosarcoma’. Clear surgical margins were confirmed, which also confirmed the effectiveness of using 3D-printed guides for the complete removal of the tumor.

Design and production

The prosthesis was designed with the collaboration of members from the Department of Orthopedics and Traumatology and the Department of Biomechanics and Tissue Engineering at Dokuz Eylül University. Designs were then fabricated by Sayan Orthopedics, Ltd, İzmir, Turkey.

**Figure 4:**
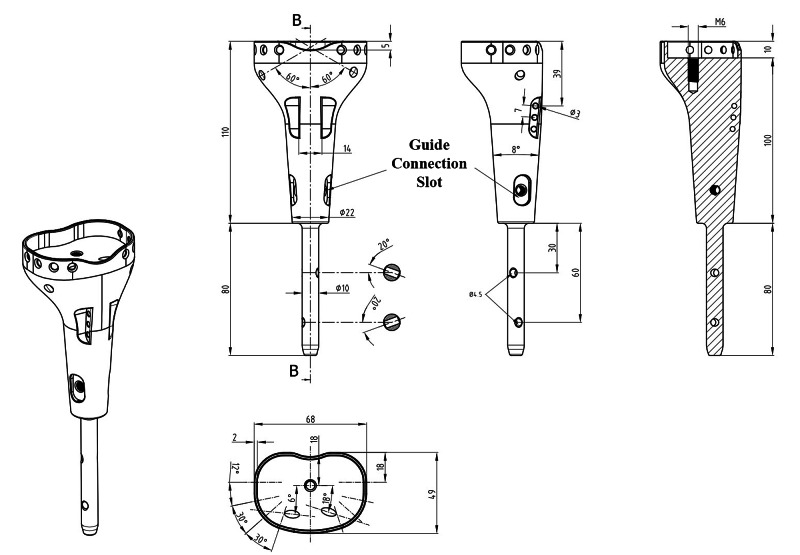
Prosthesis design The prosthesis was designed with the collaboration of members from the Department of Orthopedics and Traumatology and the Department of Biomechanics and Tissue Engineering at Dokuz Eylül University

**Figure 5:**
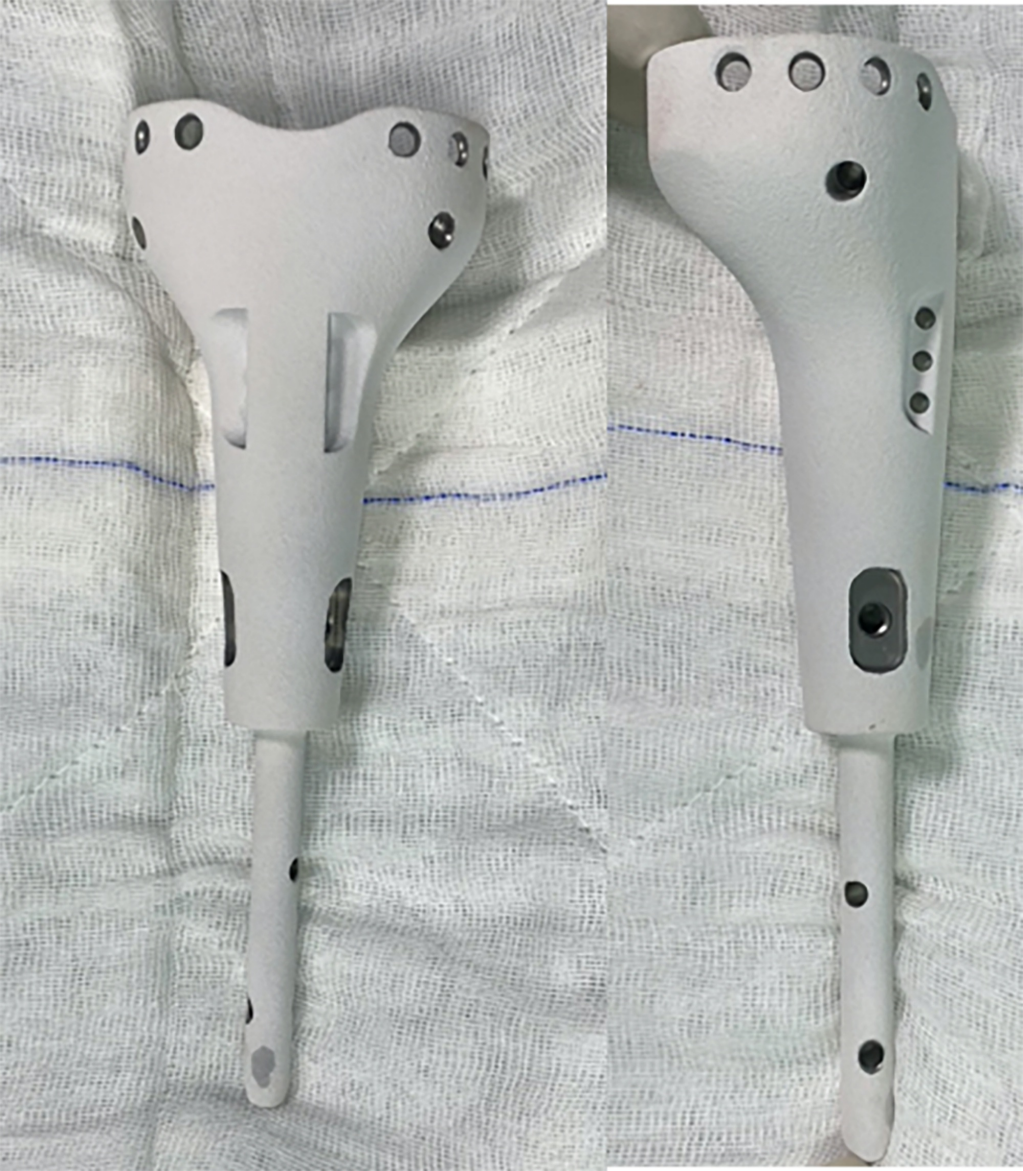
Custom-designed patient-specific tumor prosthesis

The tibial plate was designed to house and press-fit the remaining bone tissue and its attachments. Six cortical screw holes were included in the design for the fixation of bone tissue onto the prosthesis. The prosthetic body has two cortical screw locations on both sides to augment the fixation of the tibia plateau. Suture holes for fixation of the tibial tubercle were included in the prosthetic design. Thus, the continuity of the extensor mechanism will be ensured. There are two locking screw holes that fix the stem with the distal tibia. A patient-specific distal locking guide was also designed and fabricated to shorten the surgery time and minimize bone loss (Figure 4). It is aimed to enrich osteointegration of both stem and prosthetic proximal with bone by hydroxyapatite coating of both the distal stem and the prosthetic body to stimulate bone ingrowth and osteointegration [5]. The 3D-printed prosthesis was made from titanium alloy (Ti6Al4V) (ISO 5832-3) using the computer numerical control (CNC) milling method with a diameter of 70 mm (Figure 5). TI PLAZMA 280-320 micron + HA 60-90-micron coating process was done by HTI Technologies (Décines-Charpieu, France).

Custom-designed patient-specific tumor prosthesis was obtained and finite element analyzes were performed in terms of implant durability and stress effects. Virtual biomechanical evaluations, as well as finite element analysis, were performed by the Department of Biomechanics and Tissue Engineering at Dokuz Eylül University. Before clinical application, the prosthesis underwent a thorough process of cleaning and sterilization at the Dokuz Eylul University Central Sterilization Unit.

Final surgery

**Figure 6:**
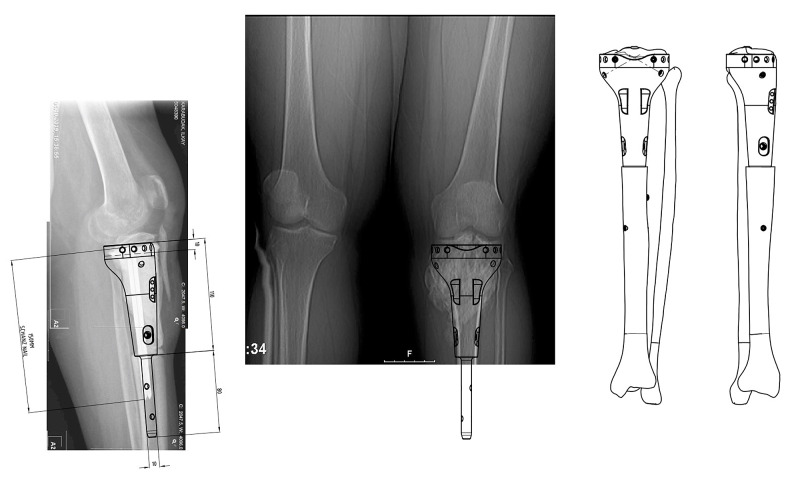
Preoperative templating

**Figure 7:**
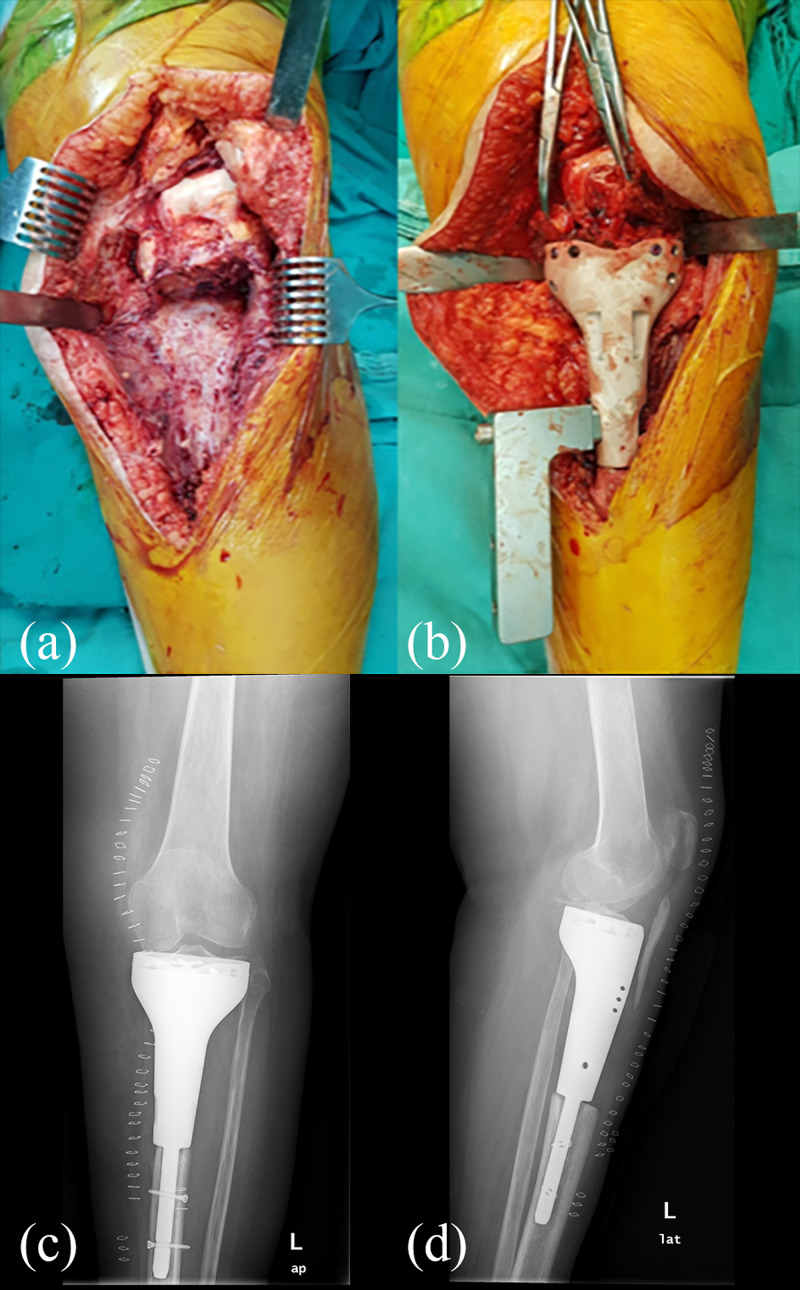
Perioperative images and postoperative X-rays (a,b) During the surgery, careful dissection of the surrounding soft tissues was ensured; the images show the implantation stage of the reconstruction; (c,d) early postoperative X-ray images

The final surgery was performed after the conclusion of preoperative templating (Figure 6) by the Chief of the Orthopedics and Traumatology Department (Dr. Hasan Havitcioglu). Careful dissection of the key structures of the knee was carried out, and the viability of the proximal tibia and its ligamentous attachments was checked by evaluating their blood flow, before proceeding with the operation. The cement spacer was removed. The tibia plateau was press-fitted to the tibial plate of the prosthesis. The distal tibia was reamed to fit the 3D-printed prosthesis stem. All stages of the procedure and positioning of the prosthesis were checked using a C-arm fluoroscopy unit. Four cortical screws parallel to the tibia plateau were inserted to enhance the stability of primary fixation. Two cortical screws were inserted diagonally towards the eminence of the tibia from the body of the prosthesis (Figure 7).

Postoperative management

The patient's knee was locked in full extension for three weeks after the operation. With physical therapy, 90 degrees of flexion was gradually achieved. Using angled immobilizers, full weight-bearing was performed four weeks after the surgery. The brace was discarded at eight weeks. By 10 months, she could walk independently without aids and pain. The follow-up X-rays showed satisfactory implant alignment. No evidence of implant loosening and tumor recurrence was seen.

Results

**Figure 8:**
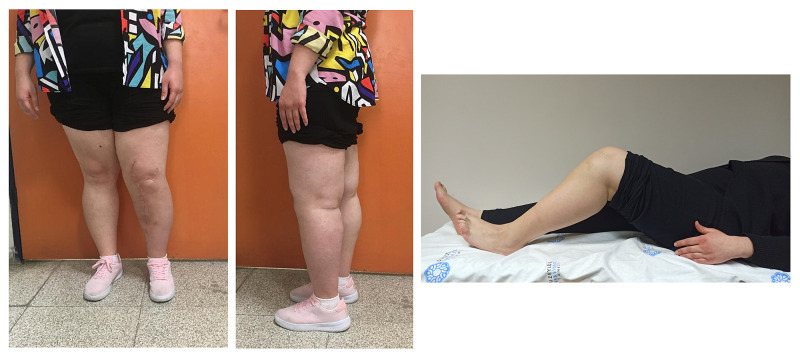
Limb function 12 months after 3D-printed prosthesis reconstruction

**Figure 9:**
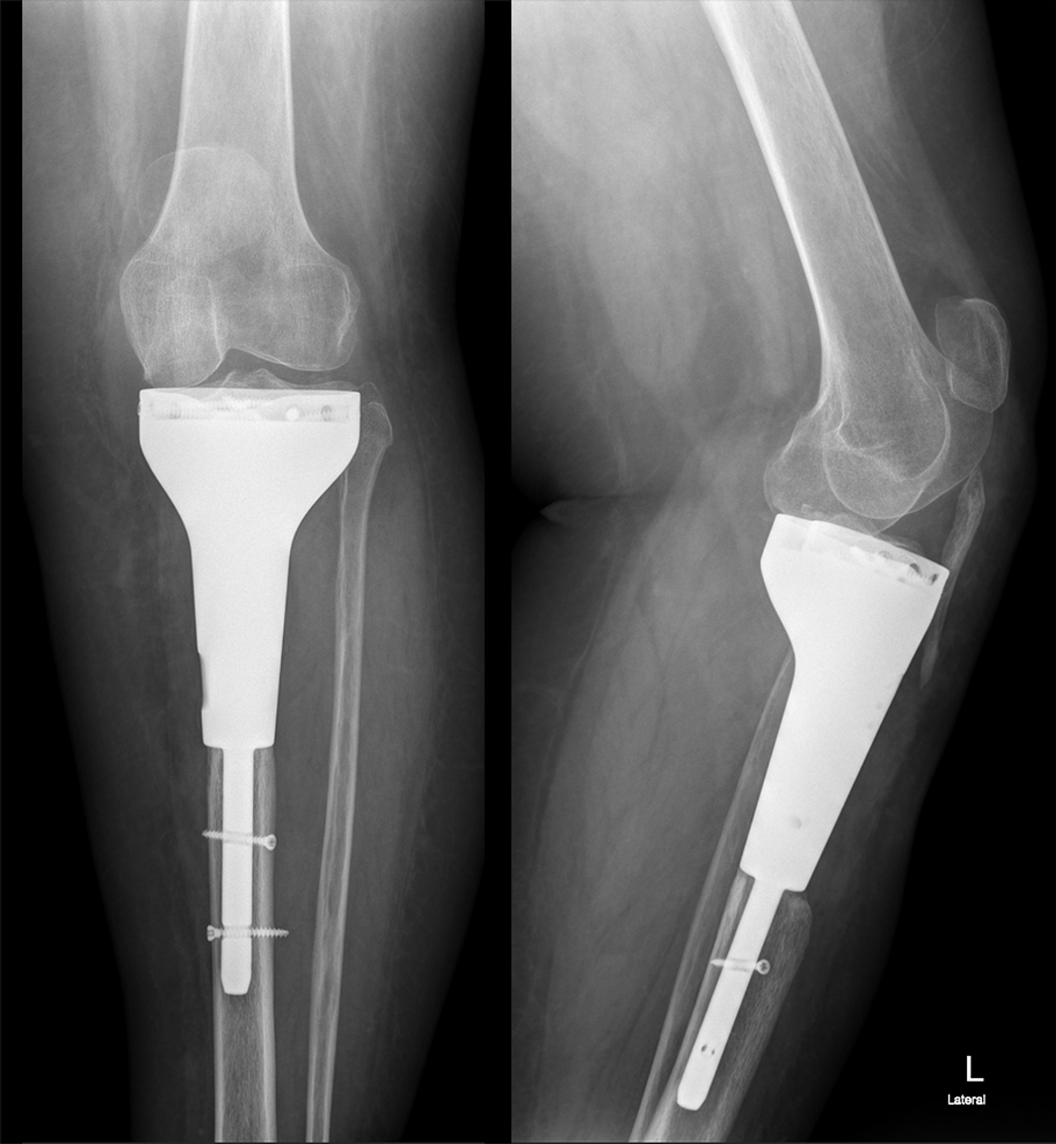
Postoperative first-year X-rays

Hydroxyapatite coating of both the distal stem and prosthetic body and the rather unusual fixation methods allowed for better stability, which in turn allowed for a healthier extensor mechanism. The range of motion was evaluated at weekly intervals and the physical therapy plan was altered accordingly to our findings; 80 degrees of flexion was attained four weeks after the procedure. We now have 100 degrees of range of motion for the affected extremity (Figure 8). No symptoms or radiological evidence of implant loosening was detected (Figure 9).

## Discussion

Treatment of massive bone defects after malignant tumor resection of long bones requires meticulous surgical technique and considerable preoperative planning, especially since the treatment options are very limited. The introduction of 3D printing has broadened the scope of many medical specialties, enhanced productivity and efficiency, and allowed surgeons to meet the anatomical and physiological needs of the pathology in hand [6-11].

Successful management of osteosarcoma can be very challenging. The oncological treatment principles are centered upon salvaging the limb without compromising the general well-being of the patient. Many factors including cost, dependency on foreign-made implants, availability, the efficiency of the implant considering the anatomical differences among sexes and ethnicity, options for revision procedures, and complications like infection, loosening, and recurrence have to be considered in the treatment algorithm.

Cost is a major factor when considering treatment options. The financial implications of the use of prostheses are thoroughly reviewed in the literature; 3D printing technology, widely available in our region, can reduce the dependency on foreign-made implants. Geographical risks and global economic recessions can cause currency depreciation and threaten the availability of the required implants. This technology can enable implant production to be done domestically. With the experience gained through the manufacturing process, we can ultimately accelerate the development of new technological advancements.

Anatomical differences among sexes and ethnicity are a new topic of discussion. Recent studies have described the anatomical variations among ethnic groups and sexes. For instance, it has been reported that Caucasians have a higher tibial torsion angle and lower varus alignment than the Japanese [12]. Differences in anthropometry between ethnic groups suggest and raise concerns that the existing femoral implant designs may not properly accommodate the needs of every ethnicity and gender [13,14]; 3D-printed and domestically produced implants address these variations by considering the patient's anatomy and bone defect and minimize the risk of implant incompatibility, loosening, patient dissatisfaction, and the need for revision surgery, thereby further lowering the total cost and its impact on the healthcare system.

Several studies have compared patient-specific instruments (PSI) with computer navigation-assisted surgery in terms of accuracy and surgery time. Studies have shown that PSIs achieved an acceptable resection accuracy with a shorter surgery time [15,16]. Additionally, 3D-printed cutting guides do not require an additional cluster of equipment and facilitating personnel, which can also help decrease the amount of OR traffic. Pre-prepared 3D-printed resection guides may also indirectly help minimize the risk of infection, even though the literature has conflicting and inconclusive reports on this topic [17,18].

3D-printed joint-preserving prosthetics, designed and produced according to the patient's needs, can decrease the need for revision surgery. Preserving the key structures for movement can result in a better sense of proprioception [2] and restore the native kinematics of the knee [19]. The prosthesis design did include a femoral stem to avoid femoral cuts and preserve native healthy bone. The cutting procedure can generate excess heat that can trigger bone necrosis and may compromise fixation and possibly increase the risk of infection [20]. The widespread adaptation of 3D-printed PSI can be challenging since the availability of such technology is scarce. However, adopting the technology for designing and manufacturing custom 3D-printed prostheses can open the door for endless designs for revision systems if need be.

## Conclusions

With this paper, we wanted to emphasize that in a select group of young patients, joint-preserving prosthetic reconstruction using 3D-printing technology can be an effective weapon in the surgeon's hands as a salvage procedure. With the mass adoption and widespread use of emerging new technologies, cost and time efficiency can be greatly optimized and technology can once again illuminate the path of medical science.
